# Exploring the relationship between older adults’ online health information seeking, negative emotions and prevention behaviors in the pandemic context: a two-wave longitudinal study

**DOI:** 10.3389/fpubh.2024.1377017

**Published:** 2024-06-12

**Authors:** Tianchang Liu, Xiaokang Song, Qinghua Zhu

**Affiliations:** ^1^School of Information Management, Nanjing University, Nanjing, China; ^2^School of Management, Xuzhou Medical University, Nanjing, China

**Keywords:** online health information seeking, older adults, SHARE, COVID-19, negative emotion, longitudinal study

## Abstract

**Introduction:**

During the COVID-19 pandemic, older adults were facing more mental health issues that may cause complex impacts on pandemic prevention, and turning to the internet for health information is a double-edged sword for them. This study aimed to investigate the reciprocal relationship between negative emotions and prevention behaviors in older adults, as well as the direct and moderating effects of online health information seeking (OHIS) on negative emotions and prevention behaviors.

**Methods:**

Based on the common-sense model of self-regulation (CSM) and a sample of more than 20,000 participants from the Survey of Health, Aging and Retirement in Europe (SHARE), this study first used an autoregressive cross-lagged panel model (CLPM) to analyze the longitudinal effect of negative emotions on prevention behaviors. Second, the study used ordinary least squares (OLS) regression to explore the influence of OHIS usage frequency changes on negative emotions and prevention behaviors. Third, the study used multigroup analysis to examine the moderating effect of OHIS usage frequency changes on the CLPM.

**Results:**

The findings indicate a significant longitudinal association where initial negative emotions predicted later prevention behaviors (*β* = 0.038, *p* < 0.001), and increased OHIS frequency was linked to positive changes in prevention behavior (*β* = 0.109, *p* < 0.001). Multigroup analysis revealed that the connection between negative emotions or increased negative emotions and prevention behaviors remained significant for those with no change or an increase in OHIS frequency but not for those with a decrease.

**Conclusion:**

This study suggested that negative emotions may drive older adults to engage more in prevention behaviors and that OHIS can augment this effect. These results underscore the importance of addressing mental health and providing reliable online health information to support older adults in managing infectious disease risks.

## 1 Introduction

The COVID-19 pandemic had a significant impact on individuals worldwide, particularly on older adults, because the age group suffered a higher prevalence, severity, and mortality rate (1, 2). Moreover, the pandemic could lead to deterioration in the mental health of older adults due to anxiety about the lethality of COVID-19, restricted access to healthcare, and disruptions to their daily routines, which may have had complex impacts on pandemic prevention and control (3–5). In addition, studies have shown that proactive actions might mitigate mental distress during the early stage of the pandemic, and the effect was greater in older adults (6). However, as the pandemic continued, the utility of this solution seemed to diminish among older adults (7), and the social isolation caused by prevention behaviors (e.g., staying distant from others outside the home) might increase the negative emotions (e.g., depression, loneliness) of older adults (8). It has also been found that the characteristics of emotional and mental health problems vary at different stages of the pandemic (9). Thus, the long-term relationship between negative emotions and pandemic prevention behaviors is complex and worthy of further study. There are already some studies focusing on negative emotions and pandemic prevention behaviors. However, current research is mostly cross-sectional and rarely considers the reciprocal relationship between negative emotions and pandemic prevention behaviors from a longitudinal perspective.

The internet is an important source of health information for older adults and plays an important role in their health decision-making and self-caring (10, 11). During the pandemic, the desire for health information increased rapidly among older adults (12). They were increasingly turning to the internet for information about the pandemic not only for meeting their information needs but also for maintaining mental connections in coping with isolation and loneliness (13). However, due to lower familiarity with information and communications technology usage, as well as worse sensory and cognitive capabilities, there are some obstacles during the health information seeking and use process that may cause negative results. First, older adults may encounter misinformation or conflicting information online, which can further increase their stress and confusion (14, 15). In addition, constant exposure to news about the pandemic and its impact can lead to information overload and information avoidance (16), which further increases negative emotions and reluctance to engage in prevention behaviors (17). Therefore, it is necessary to explore the long-term effects of OHIS on negative emotions and pandemic prevention behaviors.

For the effect of OHIS on negative emotions and pandemic prevention behaviors, Pluye et al. (18) proposed a framework of health outcomes of online consumer health information based on a systematic review that showed that OHIS affects health behaviors through cognitive impacts. Previous research has treated worry and anxiety as mediators between OHIS and prevention behaviors and has suggested that OHIS can help prevent infections but also promote anxiety and fear, which may lead to other inappropriate behaviors (19, 20). However, few studies have investigated the influence of older adults’ OHIS on other relationships, and there is a scarcity of literature exploring the change in OHIS usage frequency at different stages of the pandemic. Therefore, clarifying the reciprocal relationship between negative emotions and prevention behaviors in older adults and the long-term effect of OHIS on them has important implications for future research and public interventions. Thus, the following research questions are proposed:

Question 1: What is the long-term reciprocal relationship between older adults’ negative emotions and their pandemic prevention behaviors?

Question 2: How does changes in older adults’ OHIS usage frequency influence changes in their negative emotions and pandemic prevention behaviors?

Question 3: What is the moderating effect of OHIS usage frequency changes between negative emotions and pandemic prevention behaviors?

## 2 Related works

### 2.1 Negative emotions and prevention behaviors

**Figure 1:**
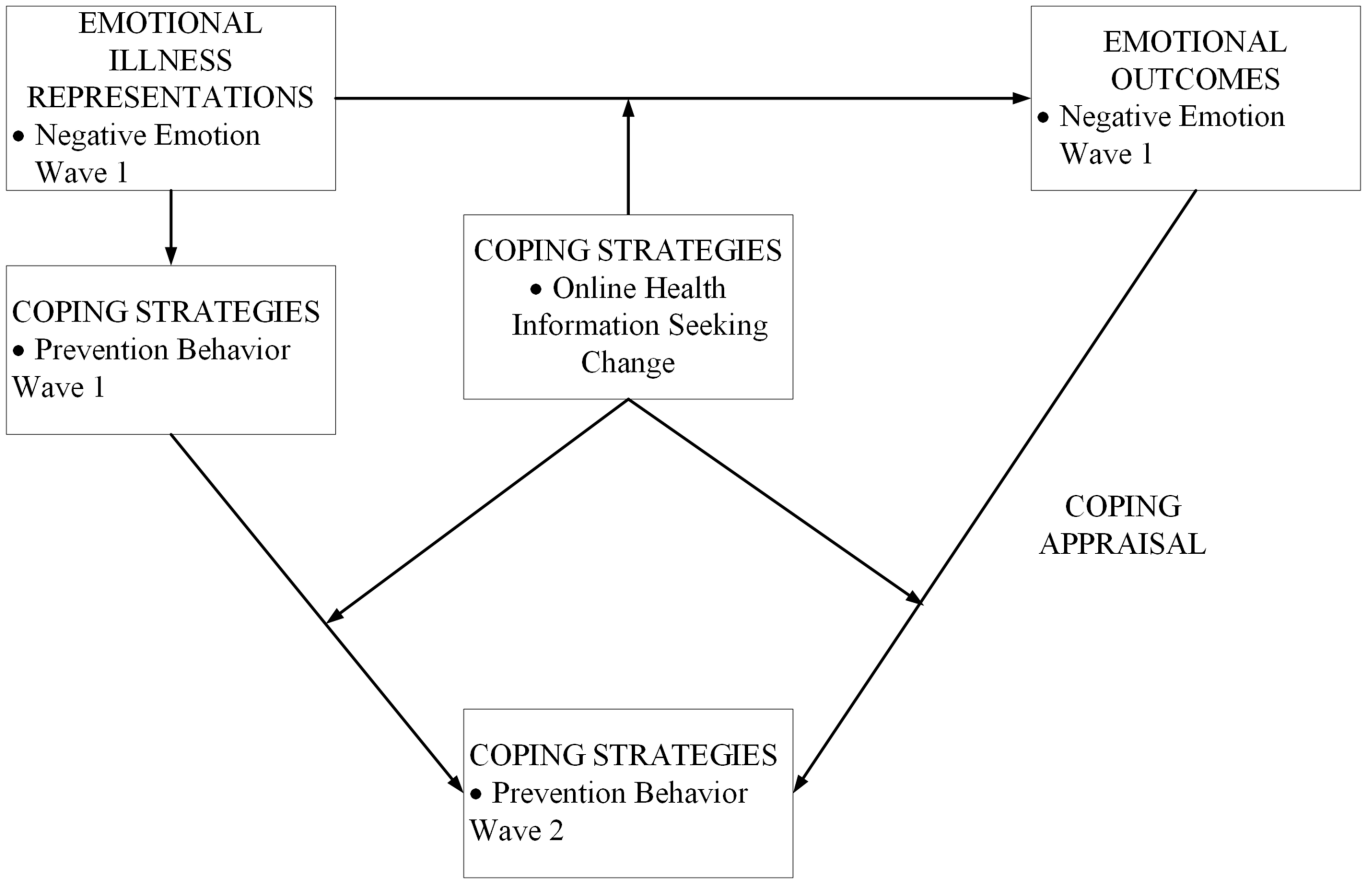
The theoretical framework of the feedback loop.

The psychological function of emotion is to motivate people to take action, and negative emotions (such as depression, nervousness and loneliness) can significantly contribute to the development or exacerbation of mental health problems (21, 22). According to theories such as the Protective Motivation Theory (PMT) (23), the Health Belief Model (24), and the Common-Sense Model of Self-Regulation (CSM) (25), negative emotion is usually related to threat perceptions and is an essential antecedent of prevention behaviors (26). In particular, CSM posits that when people are facing threats to their health, they tend to return to equilibrium by resolving the threat guided by illness representations and emotion representations (27). Moreover, CSM contains a “feedback loop” framework that describes the dynamic process by which individuals modify their emotional representations through an appraisal of coping strategies (28). In this study, coping strategies are represented by OHIS and prevention behavior, illness representation can be represented by subjective health and COVID-19 involvement, and emotional representation, which is our primary concern, can be represented by negative emotions. This study explored the relationship between emotional representation and two coping strategies. Figure 1 shows the theoretical framework of the feedback loops. Emotional illness representation directly affected coping strategies in wave 1 and indirectly affected coping strategies in wave 2 through emotional outcomes and coping appraisals. Prevention behavior in wave 1 will have an effect on prevention behavior in wave 2. As another coping strategy, OHIS can affect both negative emotion change and prevention behavior change and can moderate the relationship between negative emotion and prevention behavior by affecting coping appraisal.

Some studies have explored the relationship between negative emotions and prevention behaviors in the context of the COVID-19 pandemic. Dixon et al. (29) combined constructs from CSM, PMT, and other theories to measure adherence to prevention behaviors for 6 weeks and found worry to be a significant antecedent. Based on the knowledge-attitudes-practices model, Ning et al. (30) found that negative emotions are positively associated with COVID-19 prevention behaviors. In addition, studies using CSM have shown that negative emotions are important predictors of vaccination willingness (31), safety-seeking behaviors (32), and high levels of psychological distress, which drive people to search for health-related information online (33). In contrast, others have drawn opposite conclusions. Based on the PMT, Ezati Rad et al. (34) reported that fear negatively predicts protective behaviors. Alegria et al. (35) reported that negative emotions improve risky behaviors rather than prevention behaviors. In terms of older adults, there are fewer studies on this topic. Therefore, given the mixed findings in the literature and the characteristics of older adults, there is a knowledge gap regarding the reciprocal relationship between older adults’ negative emotions and prevention behaviors. This research aims to resolve these discrepancies from a longitudinal perspective.

### 2.2 The effect of OHIS on health levels and behaviors

Online health information seeking (OHIS) refers to individuals seeking information about their health, illnesses, and other health-prevention behaviors through the internet (36). As mentioned before, OHIS can have both positive and negative effects on older adults’ health levels and behaviors. OHISs have drawn much attention during the pandemic. According to a systematic review of the COVID-19 information seeking behavior literature, OHIS behavior is a predictor of practising prevention behavior (37). Yang and Cao (38) found that OHIS can mediate the effects of age and education on the performance of protective behaviors, which means that it is important for older adults to actively look for health information from online sources. In contrast, some studies have reported negative results. In Thailand, Yodmai et al. (39) reported that health literacy and access to health information were not significant antecedents of older adults’ COVID-19 prevention behaviors. Vismara et al. (40) reported that OHIS contributes to psychological stress and has negative effects on health behaviors during the pandemic, especially for those who have cyberchondria (negative behavior characterized by excessive online health research combined with increasing levels of health anxiety or distress). Thus, the general effect of OHIS on older adults’ negative emotions and pandemic prevention behavior is still uncertain, and there is a need to investigate the long-term effect of OHIS on older adults’ negative emotions and prevention behavior.

To address the above three research questions, the study selected data from the SHARE project with older adults aged 55 or older in 26 European countries and Israel and used an autoregressive cross-lagged panel model (CLPM) and ordinary least squares (OLS) for data analysis.

## 3 Methods

### 3.1 Samples

The data used in this study are from the “Survey of Health, Aging and Retirement in Europe” (SHARE) project (41), which is the largest European social science panel study and has been widely used in gerontology. The sampling design for SHARE involves a complex multistage process that ensures the representativeness of the sample across participating countries, including random sampling and stratification, which considers different regions or population subgroups in the sample. Which is the largest European social science panel study. Specifically, we used data from the SHARE Wave 8 survey (October 2019–March 2020) (42, 43), the first SHARE Corona Survey (June–August 2020), and the second SHARE Corona Survey (June 2021–August 2021) (44, 45). The regular SHARE was a longitudinal survey fielded every 2 years via computer-assisted personal interviewing, and the SHARE Corona Survey was collected via computer-assisted telephone interviews on pandemic-related topics.

This study selected samples of people older than 55 years old who participated in both waves of the SHARE Corona Survey and removed those samples with missing values for key variables. The final samples are from 26 European countries and Israel. The first SHARE Corona Survey was treated as Wave 1, and the second SHARE Corona Survey was treated as Wave 2.

The surveys were finished at the end of two pandemic peaks. According to the Oxford Coronavirus Government Response Tracker (OxCGRT) (46), the average number of new deaths per million and the stringency index of governmental policy responses to the pandemic in the countries during the first survey were 0.290 and 48.824, respectively, and changed to 0.696 and 45.211, respectively, during the second survey, which indicates relatively similar pandemic severity.

### 3.2 Measures

Table 1 shows the descriptions of the variables used in our research. Prevention actions and negative emotions are measured in both Wave 1 and Wave 2. Increased Negative Emotions in Wave 2 The OHIS frequency change is measured in Wave 2. The study also included several covariates, including sex, age, education level, living alone, household income, and employment, as demographic variables and subjective health, chronic disease status, and COVID-19 status as health indicators. According to previous studies, these variables have a significant influence on older adults’ OHIS behavior and prevention behavior (47, 48). The study also considered the context regarding the evolution of the pandemic and associated public health guidance during the measurement period by adding two covariates: New Deaths per Million and Stringency Index, adapted from OxCGRT. The measurements of prevention actions and negative emotions and COVID-19 incidence were adapted from previous studies (7, 30, 49–51).

**Table 1:** Description of key variables.

Name	Source/description	Values
OHIS frequency change	Source: the second SHARE Corona Survey Description: Usage frequency of internet for finding information on health-related issues since the outbreak compared with before the outbreak.	Response options were “not at all,” “less often,” “about the same,” “more often.” Divided into four groups.
Prevention actions	Source: the first and second SHARE Corona Survey Description: The number of prevention actions, including: (1) always or often staying distanced from others outside the home (2) covering coughs and sneezes (3) not meeting with more than 5 people from outside household (4) taking drugs for coronavirus prevention.	Ranging from 0 to 4.
Negative emotions	Source: the first and second SHARE Corona Survey Description: The number of negative emotions, including four types of negative emotions: nervous, depressed, lonely, trouble with sleep. Each was measured by a question about whether they felt nervous, felt sad or depressed, had trouble with sleeping or recent change in pattern, often felt lonely in last month.	Ranging from 0 to 4.
Increased negative emotions	Source: the second SHARE Corona Survey Description: The number of increased negative emotions. Each was measured by a question about whether the emotion was less so, about the same, or more so than during the first wave	Ranging from 0 to 4.
Fear of infection	Source: the first and second SHARE Corona Survey Description: The question was” Since the outbreak of Corona, did you forgo medical treatment because you were afraid to become infected by the corona virus?”	0 for no, 1 for yes.

### 3.3 Data analysis

This study first used an autoregressive cross-lagged panel model (CLPM) (52) to analyze the relationship between negative emotions and prevention behaviors in older adults. In the CLPM, after controlling for reverse causality and the influence of auto regression, the estimated cross-lagged coefficient has a clear time-sequence relationship, which conforms to the causal inference principle of “the cause comes first and the result comes later” and excludes alternative explanations that may occur in cross-sectional studies (53).

Second, the study used ordinary least squares (OLS) regression to investigate the direct effect of changes in older adults’ OHIS behavior frequency on changes in negative emotions and prevention behaviors. The changes in negative emotions and prevention behaviors were measured by subtracting the value in wave 1 from the value in wave 2.

Third, to analyze the moderating effect of OHIS, a multiple-group approach was used in the CLPM (54, 55). The samples were divided into 4 groups according to their OHIS frequency changes. After checking the collinearity of all variables in the model, the CLPM was built in the structural equation model with robust standard error, and full information maximum likelihood estimation was used because of missing data for some covariates (56). All analyses were conducted using Stata 17.

## 4 Results

### 4.1 Demographic characteristics

The means, SDs, and Pearson correlation coefficients for the dependent variables and covariates are shown in Table 2. The final sample of this study comprised 20,894 individuals, with a mean age of 68 years and an average educational level of 3.5 years. Women constituted 56% of the participants. Only 24.7% of the participants lived alone, and 26.8% of the participants were employed. The subjective health of the participants was generally at a medium level. The participants had more than one chronic disease on average. Each sample had experience using the internet after the outbreak of the pandemic, indicating that everyone had basic IT skills.

**Table 2:** Means, SDs, and Pearson correlation coefficients for dependent variables and covariates.

Variable				PB in wave1	MP in wave1	PB in wave2	MP in wave2
	Obs	Mean	Std.dev.	R (*p*-value)			
PB in wave1	20,894	2.43	0.71	1.00			
MP in wave1	20,894	0.91	1.14	0.09 (<0.001)	1.00		
PB in wave2	20,894	1.54	0.80	0.20 (<0.001)	0.09 (<0.001)	1.00	
MP in wave2	20,894	1.03	1.21	0.06 (<0.001)	0.52 (<0.001)	0.09 (<0.001)	1.00
Education	19,489	3.56	1.29	−0.03 (<0.001)	−0.04 (<0.001)	−0.04 (<0.001)	−0.03 (<0.001)
Employed	20,894	0.27	0.44	−0.09 (<0.001)	−0.05 (<0.001)	−0.09 (<0.001)	−0.06 (<0.001)
Gender	20,894	0.44	0.50	−0.09 (<0.001)	−0.18 (<0.001)	−0.08 (<0.001)	−0.17 (<0.001)
Subjective health	20,894	3.04	0.94	−0.06 (<0.001)	−0.25 (<0.001)	−0.08 (<0.001)	−0.33 (<0.001)
Married	19,489	0.75	0.43	0.01 (0.09)	−0.15 (<0.001)	−0.02 (0.03)	−0.14 (<0.001)
Age	20,894	68.00	7.34	0.05 (<0.001)	0.00 (0.87)	0.02 (<0.001)	0.01 (0.05)
Chronic disease	20,894	1.35	1.35	0.05 (<0.001)	0.16 (<0.001)	0.06 (<0.001)	0.18 (<0.001)
Covid-19 involvement	20,894	1.12	1.21	0.03 (<0.001)	0.04 (<0.001)	0.01 (0.07)	0.06 (<0.001)
New deaths per million wave 1	20,894	0.26	0.28	0.00 (0.73)	0.05 (0.00)	0.04 (<0.001)	0.04 (<0.001)
New deaths per million wave 2	20,894	0.52	0.44	−0.02 (0.03)	0.02 (<0.001)	−0.07 (<0.001)	−0.01 (0.10)
Stringency index wave 1	20,894	46.02	9.45	0.02 (<0.001)	0.01 (0.17)	0.06 (<0.001)	−0.02 (<0.001)
Stringency index wave 2	20,894	49.35	9.11	−0.01 (0.18)	0.03 (<0.001)	−0.04 (<0.001)	−0.01 (0.04)

MP, negative emotions; PB, prevention behaviors.

For the dependent variables, each participant had an average of more than one prevention behavior and negative emotion. The average number of prevention behaviors decreased by 36.49% from wave 1 to wave 2, but the average number of negative emotions increased by 33.22%. There could be possible explanations for this finding. The initial wave of the pandemic engendered a heightened awareness and sense of urgency for older adults to engage in prevention behaviors, but as time went on, the constant stress and the decreasing stringency index of the pandemic may have led to a decline in the motivation to continue engaging in prevention behaviors. In addition, the increase in negative emotions could also be due to the prolonged impact of the pandemic, especially among those who were already vulnerable due to preexisting conditions or limited access to healthcare resources.

The correlations between negative emotions and prevention behaviors across the two waves were significant (0.52 and 0.20, respectively), which implied the consistency and variability of the dependent variables over time. There were also significant positive correlations between negative emotions and prevention behaviors across waves.

### 4.2 Cross-lagged models of negative emotions and prevention behaviors

**Figure 2:**
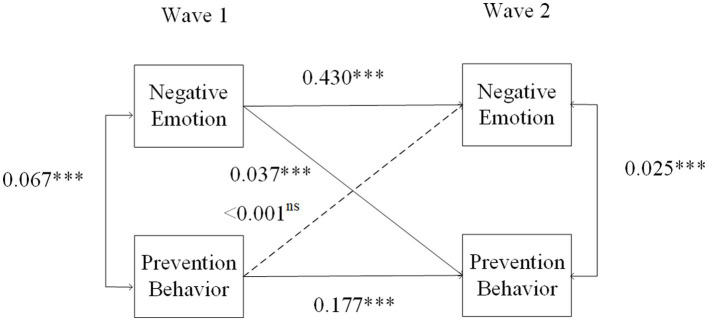
Autoregressive cross-lagged panel model of negative emotions and prevention behaviors. Covariates are not shown for clarity ns, nonsignificant; *N* = 20,894, ^*^p < 0.05, ^**^p < 0.01, ^***^p < 0.001.

Figure 2 illustrates the results of the CLPM. One-way arrows indicate the stability of constructs over time (i.e., autoregressive effects) and cross-lagged (i.e., reciprocal) effects, while two-way arrows indicate correlations. The relationship between older adults’ negative emotions and prevention behaviors was examined. First, the autoregressive coefficients of negative emotions and prevention behaviors were both significant (*β* = 0.430, *p* < 0.001 and *β* = 0.177, *p* < 0.001, respectively), but the autoregressive coefficient of prevention behaviors was less stable. Second, more negative emotions were significantly correlated with an increase in prevention behaviors in both Wave 1 and Wave 2 (*β* = 0.067, *p* < 001; *β* = 0.025, *p* < 0.001, respectively). Third, there was a significant cross-lagged effect of negative emotions in Wave 1 on prevention behaviors in Wave 2 (*β* = 0.038, *p* < 0.001), but prevention behaviors in Wave 1 had no significant effect on negative emotions in Wave 2 (*β* < 0.001, *p* = 0.823). Thus, for the first research question, it can be inferred that the negative emotions of older adults can positively predict their prevention behaviors, but not vice versa.

The fit statistics and parameter estimates of the CLPM are reported in Supplementary material A1. The β and Z values of the relationship between negative emotions in wave 1 and prevention behaviors in wave 2 (0.038) are weaker than those between increased OHIS, subjective health, employment, gender and the stringency index but are greater than those between income, new deaths per million and other variables.

**Figure 3:**
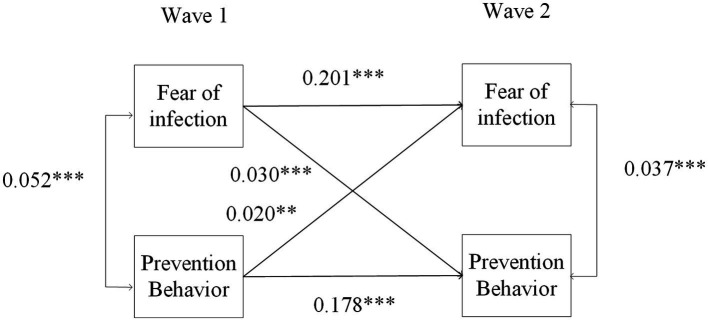
Autoregressive cross-lagged panel model of fear of infection and prevention behaviors. Covariates are not shown for clarity ns, nonsignificant, *N* = 20,894, ^*^p < 0.05, ^**^p < 0.01, ^***^p < 0.001.

To check the validity of the results, the study further examined the relationship between fear of infection and prevention behavior to better explain the results. Fear of infection is also an important emotional representation in CSM (28). The measurement of “Fear of infection” was slightly different from that of other negative emotions, so it was tested in the CLPM separately. The question was “the outbreak of Corona, did you forgo medical treatment because you were afraid to become infected by the corona virus?” The results in Figure 3 show that there is a reciprocally significant relationship between fear of infection and prevention behaviors (*β* = 0.030, *p* < 0.001; *β* = 0.020, *p* = 0.002, respectively). Thus, the results prove that fear of infection can promote prevention behaviors, but prevention behaviors also increase fear of infection among older adults (57).

**Figure 4:**
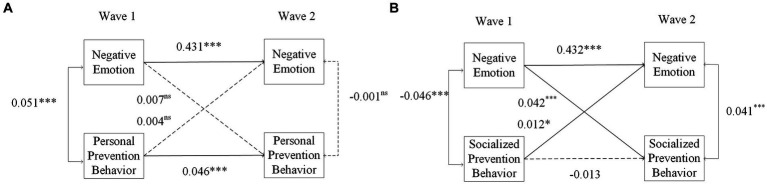
Autoregressive cross-lagged panel model of negative emotions and personal/socialized prevention behaviors. Covariates are not shown for clarity. ns, nonsignificant, *N* = 20,894, *p < 0.05, **p < 0.01, ***p < 0.001.(Model A: Personal Prevention Behavior, Model B: Social Prevention Behavior).

In addition, to better explain the discrepancies in the previous literature, this study further divided prevention behaviors into two types: personal prevention behaviors (covering coughs and sneezes; taking drugs for coronavirus prevention) and socialized prevention behaviors (always or often staying distanced from others outside the home; not meeting with more than 5 people from outside the household). Figure 4 shows that there is no significant relationship between negative emotions and personal prevention behavior and vice versa (*β* = 0.007, *p* = 0.348; *β* = 0.004, *p* = 0.543, respectively), while there is a significant bidirectional relationship between negative emotions and socialized prevention behavior (*β* = 0.042, *p* < 0.001; *β* = 0.012, *p* = 0.035, respectively). Thus, the research reveals that socialized prevention behaviors are more strongly associated with negative emotions. In the early stage, there is a negative association, but it becomes positive in the later stage and in the long term. However, for personal prevention behavior, the association turned from positive to none. This finding is in line with previous studies showing that older adults do not suffer from the negative effect of confinement measures (58), but as the pandemic continues, low-cost and habituating behavior increases linearly, while adherence to high-cost and sensitizing behaviors decreases, which may be caused by the fatigue of the pandemic and restrictive policies (59). According to CSM, we can also infer that socialized behavior is more inclined to involve feedback on emotional representations and affect future prevention behaviors.

### 4.3 The direct effect of OHIS on prevention behaviors and negative emotion changes

Table 3 shows the results of the OLS estimates for the impact of OHIS on prevention behavior change and mental health change. Only increased OHIS had a significant positive impact on changes in prevention behavior (*β* = 0.109, *p* < 0.001). In addition, among the covariates, subjective health, education, COVID-19 severity and stringency index also had significant influences on negative emotion change, while age, subjective health, COVID-19 severity, marital status, stringency index and number of new deaths per million people had significant influences on prevention behavior change. The coefficient between increased OHIS and prevention behavior change (*β* = 0.0.109) was greater than that for other variables, and for negative emotion change, subjective health had the strongest influence (*β* = −0.132). The significance of COVID-19 involvement and subjective health are in accordance with CSM, in that illness representation can influence both emotional representation and coping strategies (25).

**Table 3:** Ordinary least squares estimate for the impact of OHIS on prevention behavior change and negative emotion change (*n* = 19,489).

Dependent variable	Negative emotion change		Prevention behavior change	
	β (SE)	*p* value	β (SE)	*p* value
Decreased OHIS	0.050 (0.035)	0.152	−0.024 (0.029)	0.394
The same OHIS	0.016 (0.022)	0.466	−0.024 (0.018)	0.178
Increased OHIS	0.004 (0.024)	0.877	0.109 (0.020)	0.000
Age	0.001 (0.001)	0.301	−0.003 (0.001)	0.011
Gender	0.004 (0.017)	0.825	0.023 (0.014)	0.096
Subjective health	−0.132 (0.010)	0.000	−0.024 (0.008)	0.002
Income	0.000 (0.000)	0.533	0.000 (0.000)	0.222
Education	0.019 (0.007)	0.004	−0.001 (0.005)	0.793
Chronic disease	−0.003 (0.007)	0.637	0.000 (0.005)	0.931
Employment	0.013 (0.022)	0.564	−0.029 (0.019)	0.119
Covid-19 involvement	0.021 (0.007)	0.002	−0.014 (0.006)	0.013
Married	0.013 (0.020)	0.503	−0.057 (0.016)	0.000
Stringency index wave 2	−0.002 (0.001)	0.006	0.003 (0.001)	0.000
New Deaths per million wave 2	0.007 (0.019)	0.730	0.055 (0.016)	0.001
Constant	0.050 (0.035)	0.152	−0.881 (0.096)	0.000

### 4.4 The moderating effect of OHIS between negative emotions and prevention behaviors

**Figure 5:**
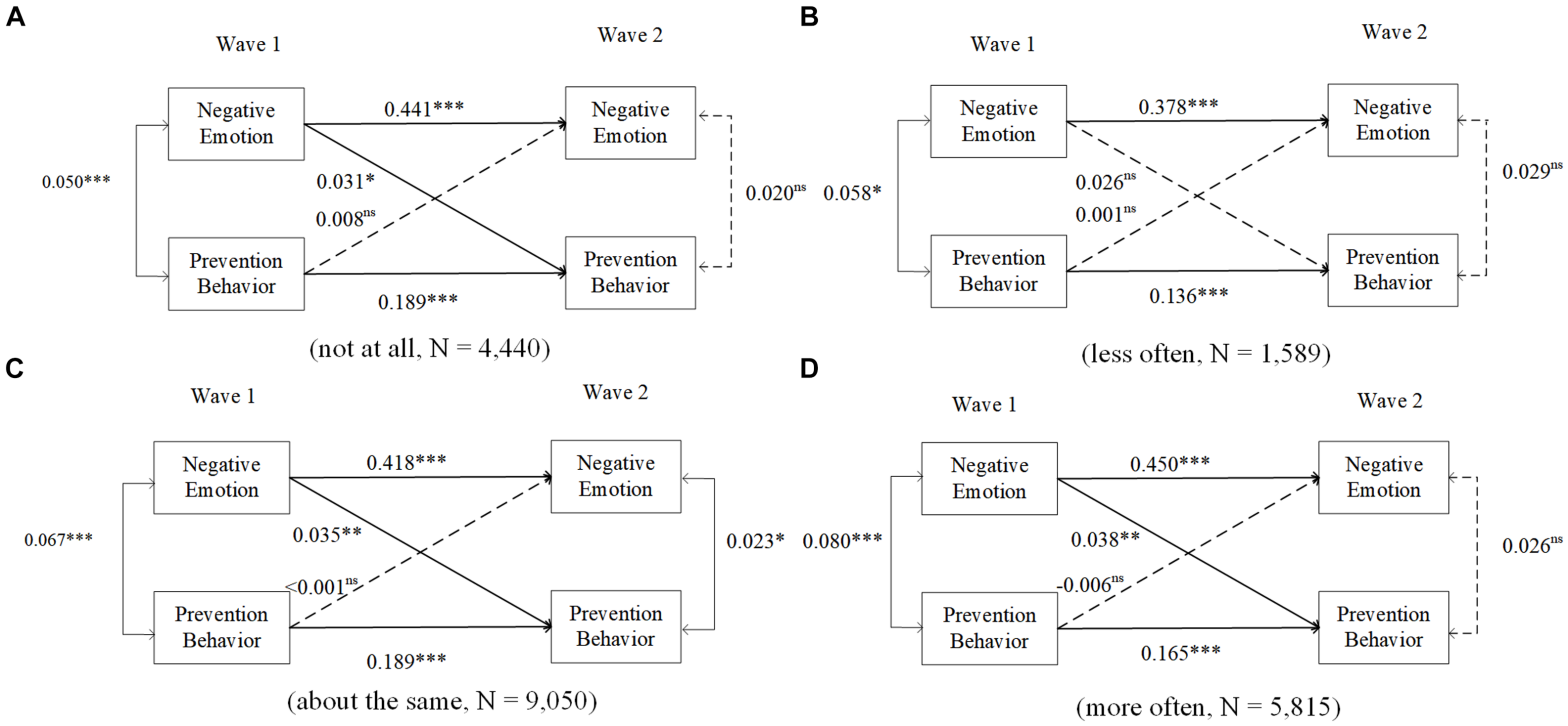
The moderating effect of OHIS between negative emotions and prevention behaviors. Covariates are not shown for clarity. ns: nonsignificant, ^*^p < 0.05, ^**^p < 0.01, ^***^p < 0.001. Models A-D are for different changes in OHIS behavior frequency (Model A: not at all, Model B: less often, Model C: approximately the same, Model D: more often).

In Figure 5, Models A-D are for different changes in OHIS behavior frequency (Model A: not at all (*N* = 4,440), Model B: less often (*N* = 1,589), Model C: approximately the same (*N* = 9,050), Model D: more often (*N* = 5,815)). There was no measurement invariance between the groups. The cross-lagged effect of negative emotions in Wave 1 on prevention behaviors in Wave 2 was significant in Models 1, 3 and 4 (negative emotions had a positive effect on prevention behaviors; *β* = 0.031, *p* = 0.045; *β* = 0.035, *p* < 0.001; *β* = 0.038, *p* = 0.003, respectively) but was not significant in Model 2 (*β* = 0.026, *p* = 0.306).

In terms of the coefficients in the models, all the data are reported in Supplementary material A2. The results are similar to the results in Supplementary material A1. To increase the validity of the results, this study also examined the moderating effect of OHIS on the relationship between increased negative emotions and prevention behaviors, as shown in Table 4. The results among the different groups are similar to the results mentioned above.

**Table 4:** Grouped regression estimate for the moderating effect of OHIS on the relationship between increased negative emotions and prevention behaviors (*n* = 19,489).

OHIS frequency change	Association between increased negative emotion and prevention behavior change	
	β (SE)	*p* value
No OHIS (*N* = 4,148)	−0.006 (0.023)	0.790
Decreased OHIS (*N* = 1,479)	0.038 (0.036)	0.282
The same OHIS (*N* = 8,438)	0.048 (0.015)	<0.001
Increased OHIS (*N* = 5,424)	0.073 (0.016)	<0.001
All (*N* = 19,489)	0.053 (0.009)	<0.001
All, OHIS controlled (*N* = 19,489)	0.047 (0.009)	<0.001

Therefore, for the third research question, we can conclude that a change in OHIS frequency can moderate the association between negative emotions and preventive behaviors in older adults.

## 5 Discussion

### 5.1 Key findings

This study investigated the reciprocal relationship between negative emotions and prevention behaviors in older adults, as well as the direct and moderating effects of older adults’ OHIS behavior on negative emotions and prevention behaviors, based on SHARE data.

The results indicate that negative emotions can positively predict the prevention behaviors of older adults. This finding is in line with previous studies showing that anxiety is associated with the prevention behaviors of older adults (7, 48). However, a previous study also showed that fear did not predict prevention behavior 6 months later in US women (60), possibly because older adults have more life experiences and tend to become more cautious and inclined to have better compliance with preventive measures after experiencing negative psychological emotions (61). Moreover, the cross-lagged effect between prevention behavior in wave 1 and negative emotions in wave 2 was not significant. The results again indicate that adhering to prevention behavior is not a sufficient solution to older adults’ mental health issues, and further coping strategies are needed. In terms of the magnitude of the relationship, increased OHIS, subjective health, employed, gender, and stringency index have higher coefficients. The results suggest that the impact of negative emotions might be relatively moderate compared to that of these other influential factors. In addition, it reflects the multidimensional nature of factors that influence health behaviors, including emotional, socioeconomic, contextual, and informational factors. This study also revealed that the discrepancies in previous literature are possibly caused by differences in certain behaviors studied. Many previous studies treated prevention behaviors as an aggregate variable (30, 34, 35), or as a single behavior (31, 32), but in this study we found that negative emotions can have a greater influence on socialized prevention behavior than on personalized behavior. This finding is in line with the study by Dixon et al. (29), which revealed that anxiousness is associated with physical distancing but not hand washing or face covering.

The OLS results show that compared with no OHIS, only increased OHIS has a significant positive impact on prevention behavior change. A possible explanation is that older adults with increased OHIS can obtain information and methods related to prevention, and in combination with their concern for their own health, they will be more proactive in prevention. In contrast, although the information obtained by OHIS related to the pandemic may enable older adults to understand the pandemic and reduce panic, reports on its serious consequences may also increase anxiety. However, the study revealed that subjective health had the strongest correlation with negative emotion change, showing a negative association. This finding is consistent with a previous study (62). This study also revealed no significant effect of OHIS on older adults’ negative emotions, which is consistent with the findings of a previous study suggesting that OHIS in older adults may stem from their anxiety as they strive to find information about themselves or social relationships (63). Therefore, OHIS can significantly enhance prevention behaviors but has no significant effect on alleviating negative emotions, which are more strongly associated with physical health. The discrepancy among previous studies regarding the influence of OHIS on prevention behaviors was possibly caused by the measurement of OHIS [e.g., the existence of OHIS (38), access to health information (39), and excessive OHIS behavior (40)]. Few studies have investigated the changes in OHIS usage frequency over time (64). This result is consistent with a previous study showing that the likelihood of changing health behavior is positively correlated with the frequency of OHIS behavior (65).

The results of the multigroup CLPM revealed that the long-term effect of negative emotions on prevention behavior varies among older adults with different changes in OHIS frequency. With the exception of older adults with reduced OHIS, negative emotions have a significant positive influence on their prevention behaviors, with the highest among those with increased OHIS, followed by those with unchanged frequency, and the lowest among those without OHIS. This suggests that efforts to promote OHIS among older adults could be particularly important for individuals with negative emotions, as OHIS may help to promote the impact of mental health issues on prevention behaviors. In addition, the decreased OHIS among older adults may indicate the existence of information avoidance, which weakens the impact of mental health issues. Health information avoidance is a barrier to older adults’ pandemic prevention (66). The determinants of information avoidance about the COVID-19 pandemic include less negative affective risk responses, more pronounced descriptive and injunctive avoidance norms, and perceived information overload (67), so it is important to provide interventions on both information accessibility and health literacy to older adults who have less OHIS. Therefore, older adults who engage in OHIS behavior have greater access to accurate and up-to-date information about the pandemic, which can help them better understand the risks and benefits of prevention behaviors and therefore support prevention behaviors. In general, OHIS plays a moderating role in the relationship between older adults’ negative emotions and prevention behaviors, which promotes the positive impact of negative emotions on prevention behaviors.

## 6 Implications

### 6.1 Theoretical implications

Overall, our study has several theoretical implications. First, it contributes to the growing body of literature on the relationships among OHIS, mental health, and behavior, particularly among older adults. This study examines the reciprocal longitudinal relationship between negative emotions and prevention behaviors and proves that OHIS and negative emotions are both important antecedents of protection behaviors. This study also highlights the unique contribution of OHIS in moderating the relationship between negative emotions and prevention behaviors. The results reduce the doubts and confusion caused by the previous literature. Second, the study also provides a reference for other well-known predictors in the model, such as subjective health. Our results revealed that subjective health has a significant positive correlation with negative emotion and prevention behavior. For future research, the significant role of subjective well-being in our model implies the importance of considering both emotional representation and illness representation according to CSM when studying pandemic-related health behaviors and designing interventions.

### 6.2 Practical implications

The findings of this research also have several implications for healthcare professionals and policymakers who work with older adults.

First, to promote effective prevention behavior, it is crucial to consider the emotional status of older adults, and a comprehensive and personalized approach is needed to promote older adults’ mental health. According to our findings, healthcare professionals should pay special attention to older adults who report lower subjective health, as they are more likely to exhibit decreased prevention behavior and negative emotional changes.

Second, the direct and moderating effects of OHIS on prevention behavior suggest that providing older adults with access to credible health information can be an effective strategy for promoting prevention behavior, but according to previous literature, OHIS may also have negative effects, so it is crucial to prevent information avoidance and take care of those suffering from unhealthy levels of health anxiety. Thus, healthcare professionals should consider providing older adults with health information and resources that are tailored to their specific needs and preferences and continuing to combat misinformation and conflicting health information online. Policymakers should also invest in digital inclusion initiatives to bridge the digital divide among older adults, including providing affordable technology devices, improving internet connectivity, and offering digital literacy training to enhance older adults’ digital skills and confidence.

Third, in light of the study’s finding that negative emotions have a bidirectional significant positive relationship with socialized protection behaviors. Thus, healthcare campaigns can emphasize the collective aspect of protective measures. Community organizations can play a pivotal role in organizing health education programs, peer support networks, and social activities for older adults. These interventions can foster social connections, alleviate feelings of isolation, and promote engagement in preventive behaviors.

Finally, the findings from this study highlight the importance of considering the long-term effects of OHIS during public health crises. Healthcare providers and organizations should prioritize promoting OHIS behavior throughout the pandemic. Regular updates, reminders, and practical tips can help maintain older adults’ motivation to engage in protection behaviors and stay informed. In addition, policymakers and healthcare authorities should incorporate OHIS promotion as a crucial component of public health preparedness for future crises and develop proactive communication strategies to empower older adults with accurate and timely health information.

### 6.3 Limitations and future research

The study also has several limitations that have implications for future research. First, the data we used are based on self-reports and may therefore reflect a subjective perspective. Future research can consider the inclusion of secondary data such as user logs. Second, a single item rather than psychometrically sound instruments was used to measure each negative emotion and prevention behavior due to the secondary analysis of SHARE data. Thus, the specific impact paths and mechanisms have not been explored in depth, and more research can be conducted in the future by combining qualitative and quantitative analysis methods. In addition, exploring the different effects of actively and passively obtaining health information on older adults’ prevention behavior is worthwhile.

## 7 Conclusion

Based on SHARE data, this study investigated the reciprocal relationship between negative emotions and prevention behaviors in older adults, as well as the direct and moderating effects of older adults’ OHIS during the pandemic. The findings suggest that negative emotions can positively predict prevention behaviors. In addition, OHIS can have a direct effect on changes in prevention behaviors and can have a moderating effect on the relationship between negative emotions and prevention behaviors. This study enhances the understanding of older adults’ OHIS on negative emotions and pandemic prevention behaviors and demonstrates the value of fostering OHIS in resolving long-term public health crises and preventing infectious diseases.

## Data availability statement

The data analyzed in this study is subject to the following licenses/restrictions: The data that support the findings of this study are available at the SHARE Research Data Center to the entire research community free of charge (www.share-project.org). Restrictions apply to the availability of these data, which were used under license for the current study, and so are not publicly available. Requests to access these datasets should be directed to http://www.share-project.org/data-access/user-registration.html?L=.

## Author contributions

TL: Writing – original draft, Writing – review & editing, Conceptualization, Data curation, Formal analysis, Validation. XS: Writing – review & editing, Conceptualization, Methodology, Funding acquisition. QZ: Writing – review & editing, Funding acquisition, Supervision.

## Supplementary material

A1 Parameter estimates in the CLPM of negative emotions and prevention behaviors.

A2 Parameters of the four CLPMs with Different Changes in the OHIS Behavior Frequency.
